# Gut microflora may facilitate adaptation to anthropic habitat: A comparative study in *Rattus*

**DOI:** 10.1002/ece3.4040

**Published:** 2018-06-14

**Authors:** Amruta Varudkar, Uma Ramakrishnan

**Affiliations:** ^1^ National Centre for Biological Sciences Bangalore India

**Keywords:** 16s metagenomics, anthropo‐dependent, intestinal microflora, physiological plasticity, *Rattus rattus*, *Rattus satarae*

## Abstract

Anthropophilic species (“commensal” species) that are completely dependent upon anthropic habitats experience different selective pressures particularly in terms of food than their noncommensal counterparts. Using a next‐generation sequencing approach, we characterized and compared the gut microflora community of 53 commensal *Rattus rattus* and 59 noncommensal *Rattus satarae* captured in 10 locations in the Western Ghats, India. We observed that, while species identity was important in characterizing the microflora communities of the two *Rattus* hosts, environmental factors also had a significant effect. While there was significant geographic variation in the microflora of the noncommensal *R. satarae*, there was no effect of geographic distance on gut microflora of the commensal *R. rattus*. Interestingly, host genetic distance did not significantly influence the community in either *Rattus* hosts. Collectively, these results indicate that a shift in habitat is likely to result in a change in the gut microflora community and imply that the gut microflora is a complex trait, influenced by various parameters in different habitats.

## 1 INTRODUCTION

The burgeoning field of metagenomics has revealed the importance of bacterial symbionts in the health of vertebrate hosts, leading some authors to suggest that the symbionts may be called “a forgotten organ” of the host (O'Hara & Shanahan, 2006). The diversity and composition of intestinal microbiota have been linked to several host physiological functions such as nutrient processing (Hooper, Midtvedt, & Gordon, 2002), structure and function of the digestive system (Robosky et al., 2005), as well as in regulating the immune systems of the host (Hooper, 2001). Recent advances in the field of culture‐independent techniques have brought to light several hidden interactions between hosts and their intestinal microflora. The diversity and stability of such interactions have led Zilber‐Rosenberg and Rosenberg (2008) to suggest that hosts and symbionts evolve together as a unit and that rapid variations in the symbionts can have important consequences in the adaptation and evolution of hosts.

Although factors leading to dysbiosis (imbalance in the intestinal microflora, often resulting in health problems) have received much attention, relatively little is known about the factors shaping the normal symbiotic microflora. Several studies on mammals and their gut microbiomes have suggested coevolution and codiversification, implying that host phylogeny shapes the gut microflora composition (Ley, Lozupone, Hamady, Knight, & Gordon, 2008). In addition, diet can act as a strong selective force on gut microflora, contributing to diverse communities not only between host species but also within a species (Bolnick et al., 2014; Muegge et al., 2011; Nakamura et al., 2011; Schwab, Cristescu, Northrup, Stenhouse, & Gänzle, 2011). Similarly, intrinsic factors such as host genetics (Linnenbrink et al., 2013), host phylogeography (Banks, Cary, & Hogg, 2009), and presence or absence of other enteric parasites (e.g., helminths, Kreisinger, Bastien, Hauffe, Marchesi, & Perkins, 2015) also influence the diversity of gut microflora at different spatial and temporal scales. Extrinsic factors such as seasonality (Maurice et al., 2015), biogeography (Linnenbrink et al., 2013), and other local environmental features (e.g., crop rotation, Chu, Spencer, Curzi, Zavala, & Seufferheld, 2013; proximity of heterospecifics, Lankau, Hong, & Mackie, 2012) influence the gut microflora, often indirectly through diet. Evidently, the gut microflora is present at the interface of host–habitat interactions and could facilitate physiological adaptation of the host to a novel environment.

The anthropic ecosystem is one such novel environment that is exclusively and actively maintained by humans. Species which have successfully colonized this environment and are almost entirely dependent on this habitat for resources are called commensal or “anthro‐dependent” species (Hulme‐Beaman, Dobney, Cucchi, & Searle, 2016). The anthropic ecosystem is undeniably different from a species’ natural environment, and the successful exploitation of this alien habitat must certainly have imposed selective pressures on the species, thereby necessitating adaptation. One of the most important factors that attract these species to an anthropic habitat is the overabundance of anthropogenic food (O'Connor, 2013). Such a change in diet type from natural to anthropogenic has been shown to have diverse impacts on the morphology, behavior, as well as physiology of the commensal species (Bateman & Fleming, 2012; Beckmann & Berger, 2003; Riyahi et al., 2013; Yom‐Tov, Yom‐Tov, & Baagøe, 2003). In this context, the intestinal microflora of the commensal species may play a substantial role in physiological adaptation by aiding digestive plasticity. Thus, a comparison between the intestinal microbiomes of commensal and noncommensal species could reveal interesting patterns associated with this shift from the natural environment to an anthropic habitat.

Rodents are often regarded as the “global commensals” as their small body size, nocturnal behavior, omnivory, and high reproductive abilities make them some of the most successful invaders of the anthropic habitat (O'Connor, 2013). Among rodents, the species *Rattus rattus* (black rat, described as Lineage I in Aplin et al., 2011) is one of the most ubiquitous commensals on almost all continents (Musser & Carleton, 2005). The global lineage of *R. rattus* originated in south India and probably spreads westwards with the growing maritime trade, leading to the familiar name of “ship rats” (Aplin et al., 2011). In its native range of south India, *R. rattus* is the most commonly captured commensal (Srinivasulu & Srinivasulu, 2012). In this same geographic region, *Rattus satarae* (white‐bellied wood rat) is endemic to the Western Ghats mountain range (Molur & Singh, 2009). As noted in previous studies, this species is restricted to the undisturbed forest fragments of the Western Ghats (Molur & Nameer, 2008). In addition, an earlier capture‐based study indicated that *R. satarae* and *R. rattus*, though distributed sympatrically in the Western Ghats, occupy different habitats: *R. rattus* is mostly captured in villages as a commensal, and *R. satarae* is predominantly found in forests (Varudkar & Ramakrishnan, 2015).

While very few studies have documented the intestinal microflora of wild rats (Firth et al., 2014), none have examined the gut microflora of mammals in the context of commensalism. In this study, we used a comparative approach to characterize and describe the gut microflora of commensal *R. rattus* and noncommensal *R. satarae*. In addition, we will address the following questions: (1) Is the gut microflora of the two species also significantly different? Which bacterial species are differentially abundant? (2) How does geographic distance influence the similarity of gut microflora in the two species? (3) What is the effect of host genetics in shaping the gut microflora communities? (4) How do different habitat characteristics shape diversity of gut microflora: forest type and seasonality in noncommensal habitat and town size and seasonality in commensal habitat?

## 2 METHODS

### 2.1 Sampling and DNA extraction

**Figure 1:**
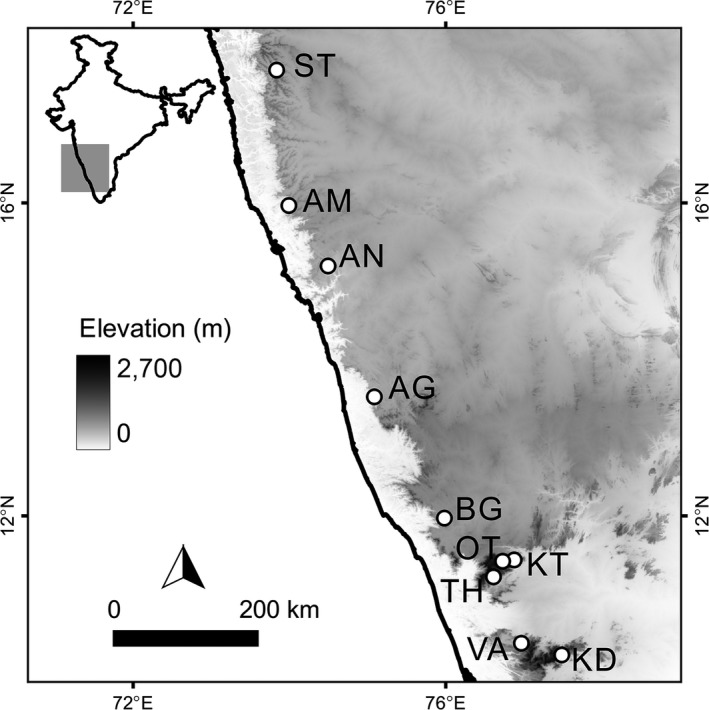
Sampling map. Locations in white circles. Sampling localities include ten villages sampled in this study (name of the forest locale in brackets): ST, Satara (Kaas plateau); AM, Amboli (Amboli reserve forest); AN, Joida (Anshi National Park); AG, Agumbe (Agumbe Rainforest); BG, Kutta (Brahmagiri Wildlife Sanctuary); OT, Ooty (Ooty Reserve Forest); KT, Kotagiri (Longwood shola); TH, Thiashola tea plantation (Thiashola); KD, Kodaikanal (Vattakanal shola) and VA, Valparai (Valparai reserve forest)

We sampled rats from 10 locations in the Western Ghats (Figure 1) following a paired sampling approach in villages and forests as described in Varudkar and Ramakrishnan (2015). We collected fresh pellets as identified by their wet appearance and soft consistency from the traps. In addition, in some sampling sites, we dissected euthanized individuals in field (euthanization with overexposure to halothane, protocol approved by the Institutional Animal Ethics committee) and collected the entire lower gastrointestinal tract (hereafter referred to as “intestinal contents”). We collected both types of samples using instruments sterilized with 70% ethanol and flamed prior to each dissection/pellet collection. We stored all biopsies as well as fecal material in 100% ethanol until transportation to the laboratory, after which, they were refrigerated at −20°C. Immediate storage in absolute ethanol prevented postcollection microbial proliferation and consequent changes in the community.

Prior to DNA extraction, we suspended the intestinal contents in 50 ml TE buffer and, after vortexing thoroughly, used 200 μl of this suspension in the next steps. We extracted DNA using QIAamp stool minikit (Qiagen, India) following the manufacturer's protocol for pathogen detection which included an additional step of heating at 95°C for 10 min during the lysis step to improve bacterial DNA concentration in the elute. We pooled, in equimolar proportions (measured spectrophotometrically), DNA elutes of gut microflora of different individuals captured from the same habitat (i.e., same species) of each location. This step assured that the gut microflora for each location was represented as a community for that location, rather than as the characteristic of an individual, minimizing noise in the data, which is most often caused by interindividual variation (Boutin, Sauvage, Bernatchez, Audet, & Derome, 2014; Hildebrand et al., 2013). Thus, there were ten pooled samples from each location for each species. Therefore, we submitted 20 pooled DNA samples to the sequencing service provider (Genotypic technologies, Bangalore) for 16s rDNA amplification and sequencing.

### 2.2 Amplicon sequencing: Illumina MiSeq

All amplicon sequencing steps were conducted by Genotypic technologies (Bangalore). Each sample was amplified with primers (S‐D‐Bact‐0341‐b‐S‐17 and S‐D‐Bact‐0785‐a‐A‐21 covering 460 bp of the V3‐V4 region, primer sequences in Klindworth et al., 2013) following the 16s Library Preparation Workflow for the Illumina MiSeq system (https://support.illumina.com/), using a high‐fidelity polymerase. Five samples were randomly selected from the 20 and were amplified again as internal library control. Each primer sequence was added at the 3′ end of an Illumina overhang adapter sequence and used to amplify ~500 bp of the targeted region of the template DNA (95°C for 3 min, 25 cycles of 95°C for 30 s, 55°C for 30 s, 72°C for 30 s, and final elongation at 72°C for 5 min). After visualization of the amplified product on an agarose gel, a second round of PCR was performed to attach indexing barcodes (same PCR conditions with eight cycles of amplification). The amplified product was quantified with an Agilent Bioanalyzer Chip (Agilent Technologies Inc., located at the sequencing service center). After the products were pooled in equimolar concentrations, SPRI (Agencourt AMPure XP®, Beckman‐Coulter) beads were used for purification and the library was validated on the Agilent Bioanalyzer. Finally, the library was sequenced on an Illumina MiSeq 300PE platform.

### 2.3 Data analysis: Quality filtering and read assembly

Upon receipt of the raw sequences, we used the FastX toolkit to visualize the data quality, specifically the quality scores, read length, per bp quality, and per read quality (Pearson, Wood, Zhang, & Miller, 1997). We merged the raw paired‐end reads with the software PEAR v 0.9.6 (Zhang, Kobert, Flouri, & Stamatakis, 2014) using a quality threshold (Phred score) of 10 for trimming the reads and detected sequence chimeras de novo using default parameters of the UCHIME algorithm (Edgar, Haas, Clemente, Quince, & Knight, 2011). Lastly, we performed a final quality filtering using a threshold of 20 (indicating an error of 1 in 100 bp) and allowing at most one “N” character in the sequence.

### 2.4 Operational taxonomic units picking, taxonomy assignment, and phylogeny

For all the procedures described in this section, we used the Quantitative Insights Into Microbial Ecology (QIIME, Caporaso, Kuczynski et al., 2010) pipeline v1.9.1. We clustered operational taxonomic units (OTUs) using the uclust algorithm (Edgar, 2010) at a 97% similarity threshold and other parameters set to default. For each OTU, we chose the longest read as the representative sequence and, using the RDP classifier v2.2 (Wang, Garrity, Tiedje, & Cole, 2007) on the Greengenes database v13.8 (DeSantis et al., 2006; McDonald et al., 2012), assigned taxonomy to all representative sequences with the following parameters: minimum 75% sequence match with confidence threshold of 0.85. We constructed rarefaction plots to determine adequacy of the 16s sequencing procedure. We aligned all representative sequences using the PyNAST algorithm (Caporaso, Bittinger et al., 2010), filtered the alignment using a lane mask file, and constructed phylogeny with FastTree (Price, Dehal, & Arkin, 2010), which was then visualized using the Topiary Explorer (Pirrung et al., 2011). Finally, we filtered reads that were unclassified, unalignable, and singletons from the OTU table.

### 2.5 Diversity indices

We performed all statistical analyses with the phyloseq package v 1.12.2 (McMurdie & Holmes, 2013) for R v 3.2.1 (R Core Team, 2015). As the total read counts were different for each sample, normalization of the library sizes was essential for comparing samples. The widely used normalization approach of resampling OTUs at the lowest read depth (Hughes & Hellmann, 2005) has been widely criticized, as through this process much informative data are discarded (McMurdie & Holmes, 2014). Instead, we used the variance stabilization method as described in Anders and Huber (2010) to transform the read counts.

To summarize alpha diversity, we calculated the following species richness estimators: phylogenetic diversity (based on phylogenetic distances between the OTUs) and Chao1 (based on number of OTUs). As these indices are highly sensitive to the presence of singletons (Hughes, Hellmann, Ricketts, & Bohannan, 2001; Lozupone & Knight, 2008), this calculation was performed before filtering the dataset for singletons, but after removing the unclassified reads. We calculated beta diversity indices, namely Jaccard (presence/absence data), Bray–Curtis (abundance data), unweighted UniFrac (phylogenetic distance between taxa), and weighted UniFrac (phylogenetic distance weighted by abundance of taxa). Further, we performed principal coordinate analysis (PCoA) and conducted an analysis of dissimilarity (“*adonis”* function in the R package “*vegan”*; Oksanen et al., 2015) on each of the aforementioned indices to compare the community composition of the two host species. We also performed a differential abundance test based on the negative binomial model as implemented in the DESeq2 package (Anders & Huber, 2010).

### 2.6 Statistical analysis: effect of geography and genetics

To investigate the effect of geographic distance on the divergence of bacterial communities, we performed multiple regression on the pairwise geographic distance matrix for all ten localities and each of the four beta diversity matrices (calculated separately for each *Rattus* host). In order to assess the effect of host genetic distance, we focused on a subset of seven locations (AN, AG, BG, OT, KT, TH, and KD, Figure 1, Table 1) that were genetically characterized in an earlier study (Varudkar & Ramakrishnan, 2015). From that study, we used the dataset for 17 microsatellite loci and calculated the Cavalli‐Sforza chord genetic distance using Microsatellite Analyzer v 4.05 (Dieringer & Schlötterer, 2003).

**Table 1:** Sample information

Location	*Rattus rattus*	*Rattus satarae*	Sample type	Forest type	Town size (Census 2011)
ST	2	3	Intestinal contents	Woody savanna	1,830
AM	1	3	Intestinal contents	Woody savanna	4,004
AG^a^	10	10	Intestinal contents	Evergreen broadleaf	500
AN^a^	2	8	Intestinal contents	Woody savanna	4,043
BG^a^	1	10	Intestinal contents	Evergreen broadleaf	1,724
OT^a^	5	6	Pellets	Evergreen broadleaf	88,430
KT^a^	10	2	Pellets	Evergreen broadleaf	28,207
TH^a^	10	4	Pellets	Evergreen broadleaf	2,106
KD^a^	10	7	Pellets	Woody savanna	36,501
VA	2	6	Pellets	Woody savanna	70,859
Total	53	59			

a Sampling locations from Varudkar & Ramakrishnan, 2015.

### 2.7 Statistical analysis: effect of habitat

To assess effect of habitat‐related features and seasonality on the diversity of gut microflora, we performed generalized linear mixed effects (GLMM) modeling on the richness estimators Chao1 and Faith's phylogenetic diversity metric (PD). The predictor variables were as follows: forest type for *R. satarae* (evergreen or woody savanna) and town size (size of human population: source, Government of India Census 2011, http://www.census2011.co.in) for *R. rattus*. Season of sampling (premonsoon or postmonsoon) was also included in the linear models for both *Rattus* species. The type of sample (intestinal contents or pellets) and the number of pooled individuals were modeled as random effects.

## 3 RESULTS

From 10 locations in the Western Ghats, we captured 112 individuals of the two species *Rattus rattus* and *Rattus satarae* (Figure 1 and Table 1). After sequencing 16S rDNA amplicons using the Illumina MiSeq platform and assembling the paired ends, quality filtering, and removing chimeras, we obtained 3,873,269 reads. From these, we detected 170,095 OTUs at the 97% similarity threshold. Although our sequencing effort was uniform across all samples and repeats, majority of the reads had very low abundance (Figure S1). Our rarefaction plots also indicated a preponderance of low‐frequency OTUs (Figure S2). Finally, after filtering the singletons, unclassified, and unalignable reads, we obtained 42,054 OTUs with an average read length of 358 bp. Of these, 12,446 OTUs were unique to *R. rattus* and 9,897 were found only in *R. satarae*.

Although different numbers of host individuals had been pooled from each location, it did not significantly affect either the number of OTUs (Figure S3a, Spearman's rank correlation coefficient ρ = 0.15, *p*‐value = .5; Mann–Whitney U test for effect of pooling more than seven individuals against pooling less than four individuals *W* = 56.5, *p*‐value = .08, tested using unfiltered data) or the compositional differences between the samples (PCoA constructed on Bray–Curtis dissimilarity matrix, Figure S3b). On the other hand, the type of the sample used (intestinal contents or pellets) had a significant effect on the number of OTUs, as pellet samples had higher mean number of observed OTUs than intestinal contents (Welch's two sample *t* test, *t* = −3.88, *df* = 22.34, *p*‐value < .01).

### 3.1 Is gut microflora of two Rattus species significantly different?

**Figure 2:**
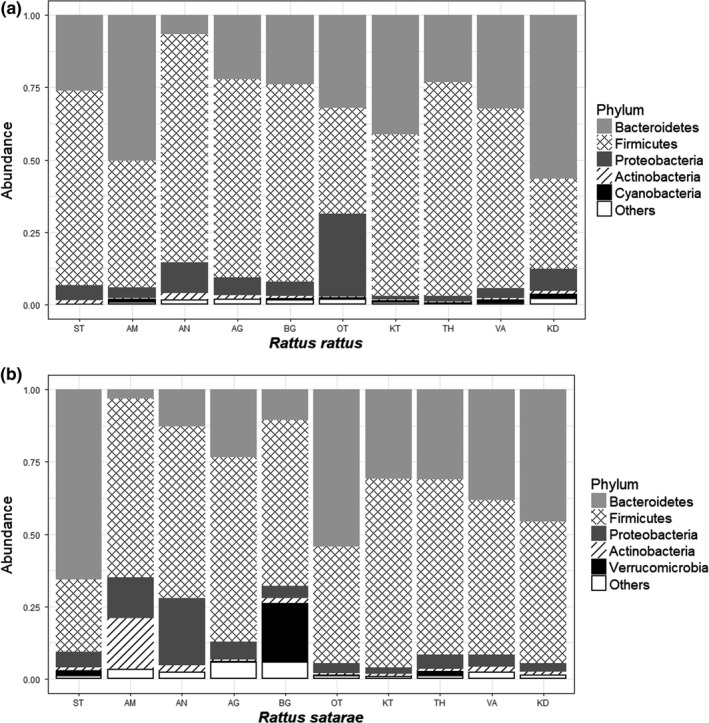
Five most common bacterial phyla in (a) *Rattus rattus* and (b) *Rattus satarae*. Abundance is the relative proportion of total operational taxonomic units s assigned to the respective phyla. Gut microflora for either *Rattus* hosts are dominated by common bacterial phyla such as Bacteroidetes, Firmicutes, and Proteobacteria

Taxonomic assignment of the OTUs revealed the presence of 25 known bacterial and two archaeal phyla. The 25 bacterial phyla were further classified into 65 classes, 104 orders, 164 families, and 263 genera. The most common phyla in both host species were Bacteroidetes and Firmicutes, followed by Proteobacteria (Figure 2). While the three phyla accounted for almost all the bacterial diversity in *R. rattus*, other bacterial phyla such as *Verrucomicrobia* were also common in *R. satarae*.

**Figure 3:**
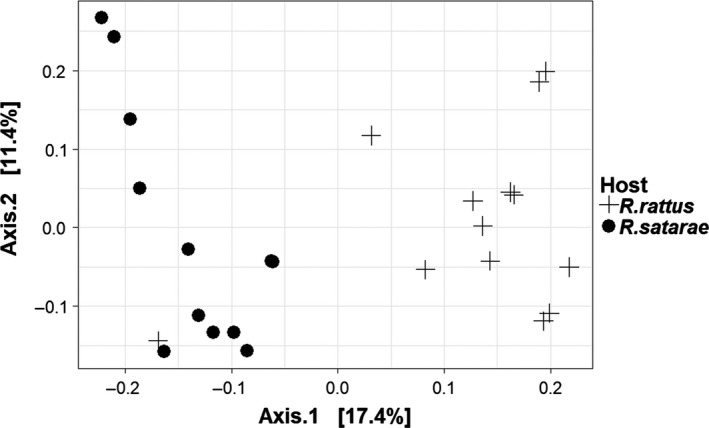
Principal coordinate analysis plot for variance‐stabilized data. Each point represents a single sample and is colored according to the host *Rattus* species. Distance between the points indicates how different their gut microflora community composition is. Plot has been constructed using first two axes of the principal coordinate analysis on the Bray–Curtis dissimilarity index (percentage values are % variation in the dissimilarity matrix explained by each axis). The plot indicates clear distinction between gut microflora communities of *R. rattus* and *R. satarae*

A principal coordinate analysis on the variance‐stabilized data indicated that the two species had distinct gut microflora compositions (Figure 3) (except for one outlier: the *R. rattus* from the KT population). Similarly, the *adonis* test indicated that the community composition was significantly different between the hosts (Jaccard *r* ^2^: .09, *p* < .001; Bray–Curtis *r* ^2^: .11, *p* < .001; unweighted UniFrac *r* ^2^: .08, *p* < .001; and weighted UniFrac *r* ^2^: .09, *p* < .05). However, the total variance explained by host species identity was low, indicating that other variables may be also influencing the community composition.

### 3.2 Which bacterial species are differently abundant?

**Figure 4:**
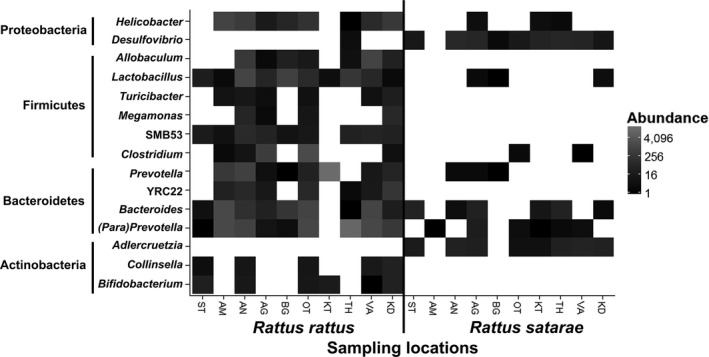
Heatmap of operational taxonomic units (OTU) differentially abundant between *R. rattus* and *R. satarae*. Each OTU is represented at least 100 times more in one species than in the other. “Abundance” indicates total count of the OUT: Darker values indicate higher abundance. Bacteroidetes and Firmicutes are highly abundant in the commensal *R. rattus*; Actinobacteria and Proteobacteria are highly abundant in the noncommensal *R. satarae*

One hundred and twenty nine OTUs were highly abundant in *R. satarae*, and 215 OTUs were highly abundant in *R. rattus* (False Discovery Rate < 0.01). Of these, Bacteroidetes and Firmicutes such as lactobacilli, *Collinsella*,* Helicobacter*, and *Bacteroides* were at least 100 times more abundant in *R. rattus*, while Proteobacteria and Actinobacteria such as *Desulfovibrio* and *Adlercruetzia* were highly abundant in *R. satarae* (Figure 4).

### 3.3 What is the effect of geographic distance and host genetics on gut microflora composition?

Mantel test results for 10 sampling locations indicated that there was no significant effect of geographic distance on the gut microflora composition for *R. rattus* for any similarity index. However, there was a significant effect of geographic distance on the gut microflora of *R. satarae* for the taxon‐based Bray–Curtis and Jaccard indices (Table 2), though not for the phylogenetic distance‐based UniFrac indices. There was no significant effect of host genetic distance on the gut microflora community in either species as indicated by both the Mantel and partial Mantel tests.

**Table 2:** Mantel tests on distance matrices for gut microflora, geographic distance, and genetic distance

Host	Distance index	Mantel test		Partial Mantel test*	
		Geographic distance	Genetic distance*	Geographic distance*	Genetic distance*
*Rattus satarae*	Jaccard	**0.39**	0.39	0.29	0.40
	Bray–Curtis	**0.38**	0.40	0.28	0.41
	Unweighted UniFrac	0.26	0.30	0.26	0.30
	Weighted UniFrac	0.28	0.31	0.39	0.32
*Rattus rattus*	Jaccard	0.03	0.23	0.12	0.26
	Bray–Curtis	0.03	0.23	0.11	0.25
	Unweighted UniFrac	0.03	0.16	0.18	0.22
	Weighted UniFrac	0.11	−0.11	0.48^a^	0.14

Bold values indicate values significant at 5% confidence level.

Tests for which a subset of seven populations was used are marked with “*”.

a Indicates significant at 10% confidence level.

### 3.4 What is the effect of habitat characteristics on gut microflora diversity?

We did not find a significant effect of habitat‐related features or the sampling season on the diversity estimates of either *Rattus* species. Neither the abundance‐based estimator “Chao1” (*R. satarae* χ^2 ^= 3.59, 2 *df*;* R. rattus* χ^2 ^= 4.33, 3 *df* compared with a null model considering only random effects: *p* > .05) nor Faith's PD (*R. satarae* χ^2 ^= 2.12, 3 *df*;* R. rattus* χ^2 ^= 1.98, 3 *df* compared with a null model considering only random effects: *p* > .05) displayed any significant correlation.

## 4 DISCUSSION

In this study, we examined the gut microflora of two *Rattus* species that occupy different habitats: namely the commensal *R. rattus* and the noncommensal *R. satarae*. In our previous study (Varudkar & Ramakrishnan, 2015), we demonstrated how this ecological differentiation resulted in dramatically different gene flow patterns for the two species across the same landscape. In this study, we report a similar divergence in the intestinal microflora communities for the two species.

Despite significant differences at the phyla‐level, the intestinal microflora community of both *Rattus* species was dominated by Bacteroidetes, Firmicutes, and Proteobacteria, which is representative of a typical murine gut microbiome (Ley et al., 2005). Host phylogeny has long been identified as a significant feature in shaping microbial communities. For instance, in a strikingly illustrative study, Ochman et al. (2010) retraced patterns of the host phylogeny in the gut microflora communities of five species of great apes. In another multispecies study on bats, it was observed that bats belonging to the same family have similar gut microflora communities (Phillips et al., 2012). In order to determine to what extent host phylogeny might influence this pattern, in addition with habitat association, a wider study on different *Rattus* species or captivity experiments could be conducted.

Although the gut microflora of different individuals was pooled, the number of pooled individuals did not have any significant effect on either the microbial diversity or the community composition (but see Hamady & Knight, 2009). The type of the sample used, whether from the intestine or from the pellets, had a significant effect, with pellets displaying higher number of bacterial OTUs than intestinal contents. It is interesting to note that the aberrant *R. rattus* gut microflora that clustered with *R. satarae* samples was from individuals captured from the borders of the village and very close to the forest. Although the unusual gut microflora of this location is inconclusive in itself, it does point toward the dynamic nature of intestinal microflora.

Similarly, microbial community composition in the noncommensal species was significantly influenced by the geographic distance between sampling locations. That this result was apparent in the taxon‐based similarity indices (such as Bray–Curtis and Jaccard) and not in the phylogenetic distance‐based metrics (weighted and unweighted UniFrac) could be reflective of the limitation of the taxonomic reference database (Parks & Beiko, 2013). It could also indicate that, although the individual taxa vary with geographic distance, their phylogenetic relationships and consequently their functional composition may remain constant (Lozupone & Knight, 2008). There was no effect of geographic distance on the gut microflora of the commensal. Host genetic distance did not affect gut microflora in either *Rattus* species. This could indicate effect of other environmental factors, beside host gene flow. Although we did not find a significant effect of habitat‐related features and sampling seasonality, we suggest that our sampling regime was limited and further detailed studies in this system might reveal interesting patterns.

The impact of diet in shaping gut microflora communities in humans and other vertebrates has been well studied (De Filippo et al., 2010; Muegge et al., 2011). Even in insects, gut microflora has been implicated in adaptation to crop rotation (Chu et al., 2013) and novel food sources (Otani et al., 2014). A large number of studies on various captive animals have revealed that perturbations in the diet result in significant changes in the gut microflora communities (e.g., parrots, Xenoulis et al., 2010; grizzly bears, Schwab et al., 2011; black howler monkeys, Nakamura et al., 2011; and elephant seals and leopard seals, Nelson, Rogers, Carlini, & Brown, 2013).

Digestive plasticity has been linked to change in biochemistry, physiology, as well as morphology of the digestive system (Green & Millar, 1987; Sabat, Novoa, Bozinovic, & Martínez del Rio, 1998). The results presented here indicate that gut microflora could also contribute to digestive plasticity and help the host to adapt to a novel dietary niche. For example, lactobacilli were abundant in the commensal species. Such a pattern was also observed in another human‐associated species, the domestic dog, which had higher lactobacilli counts than wolves (Pallin, 2012). Lactobacilli are known to increase in the presence of lactose sugar, which is an important component of milk (Daly et al., 2014), and thus, abundant lactobacilli may be a dietary adaptation to dairy‐based foods in the commensal habitat. Similarly, a high abundance of bifidobacteria has also been observed in other human‐associated domestic animals (Lamendella, Santo Domingo, Kelty, & Oerther, 2008). Bifidobacteria are implicated in enhancing digestive capabilities in humans (Mitsuoka & Kaneuchi, 1977) and could also aid commensal species in a similar manner. On the contrary, the actinobacterial genus *Adlercruetzia* was abundant in the noncommensal. In a study of the desert woodrat (*Neotoma lepida*) gut microflora, the abundance of this species was observed to decrease upon captivity for 6 months, indicating that it was possibly correlated with some factor in the natural habitat (Kohl & Dearing, 2014). Thus, an examination of particular bacterial species associated with the two hosts could indicate how the gut microflora may aid in adaptive plasticity.

Lastly, such studies on gut microflora of commensal rats have important significance from the public health perspective. In this study, human pathogens such as *Brucella*,* Chlamydia*,* Clostridium*,* Staphylococcus*, and *Rickettsia* were observed in the commensal host gut microflora. Rodents are well known as reservoirs for zoonotic pathogens (Firth et al., 2014), and studies which assess the presence of potential pathogens in commensal species may be designed especially in small towns such as those found in this study area.

## CONFLICT OF INTEREST

The authors declare no conflict of interests.

## AUTHOR CONTRIBUTION

This study constitutes part of the doctoral thesis of AV. Field sampling, molecular work, and analysis was performed by AV. UR provided funding support, laboratory facilities, and intellectual and scientific guidance.

### DATA AVAILABILITY

Primary data including sequences and sample locations have been deposited in the Dryad Repository. (https://doi.org/10.5061/dryad.5gj7056).
