# Supplementary figures and images for: Gut microflora may facilitate adaptation to anthropic habitat: A comparative study in Rattus
Source: Ecol Evol. 2018 Jun 14;8(13):6463–72. doi: 10.1002/ece3.4040 (PMC6053588; doi:10.1002/ece3.4040)

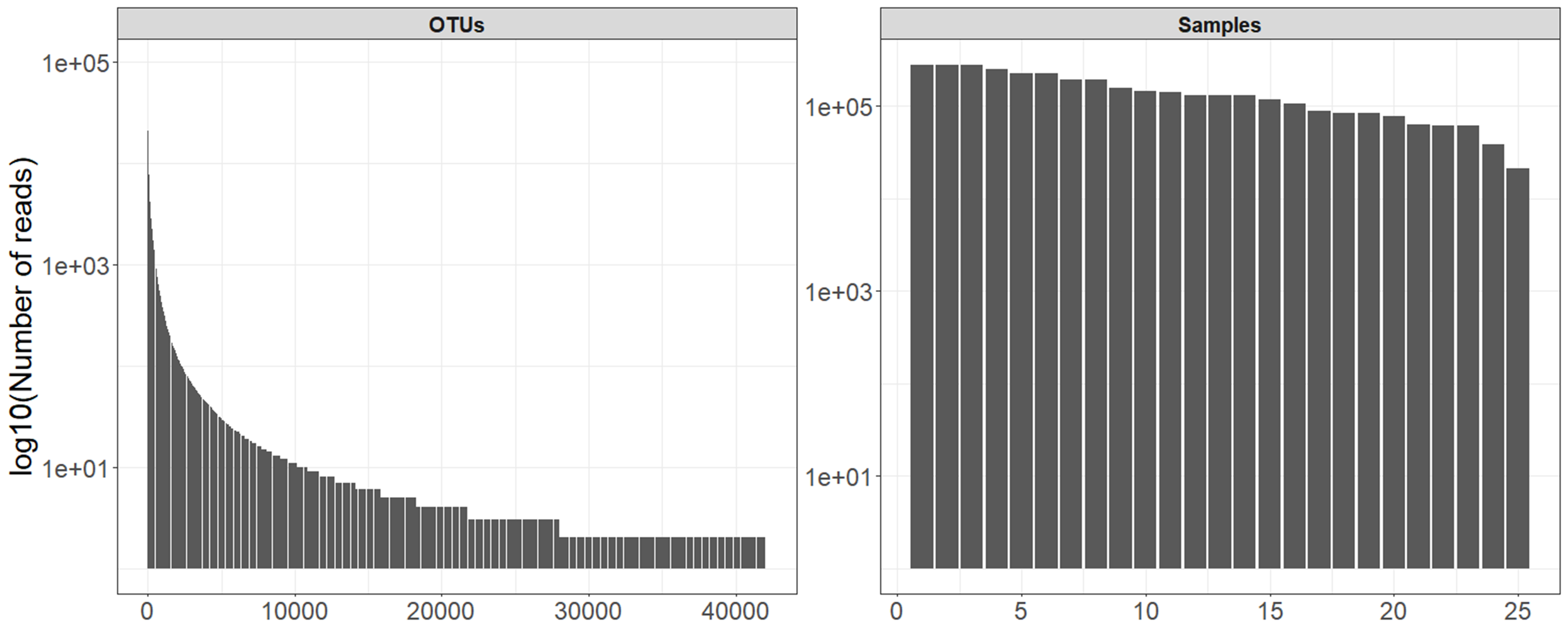

Supplement: Supplementary file 1 [file ECE3-8-6463-s001.tiff]

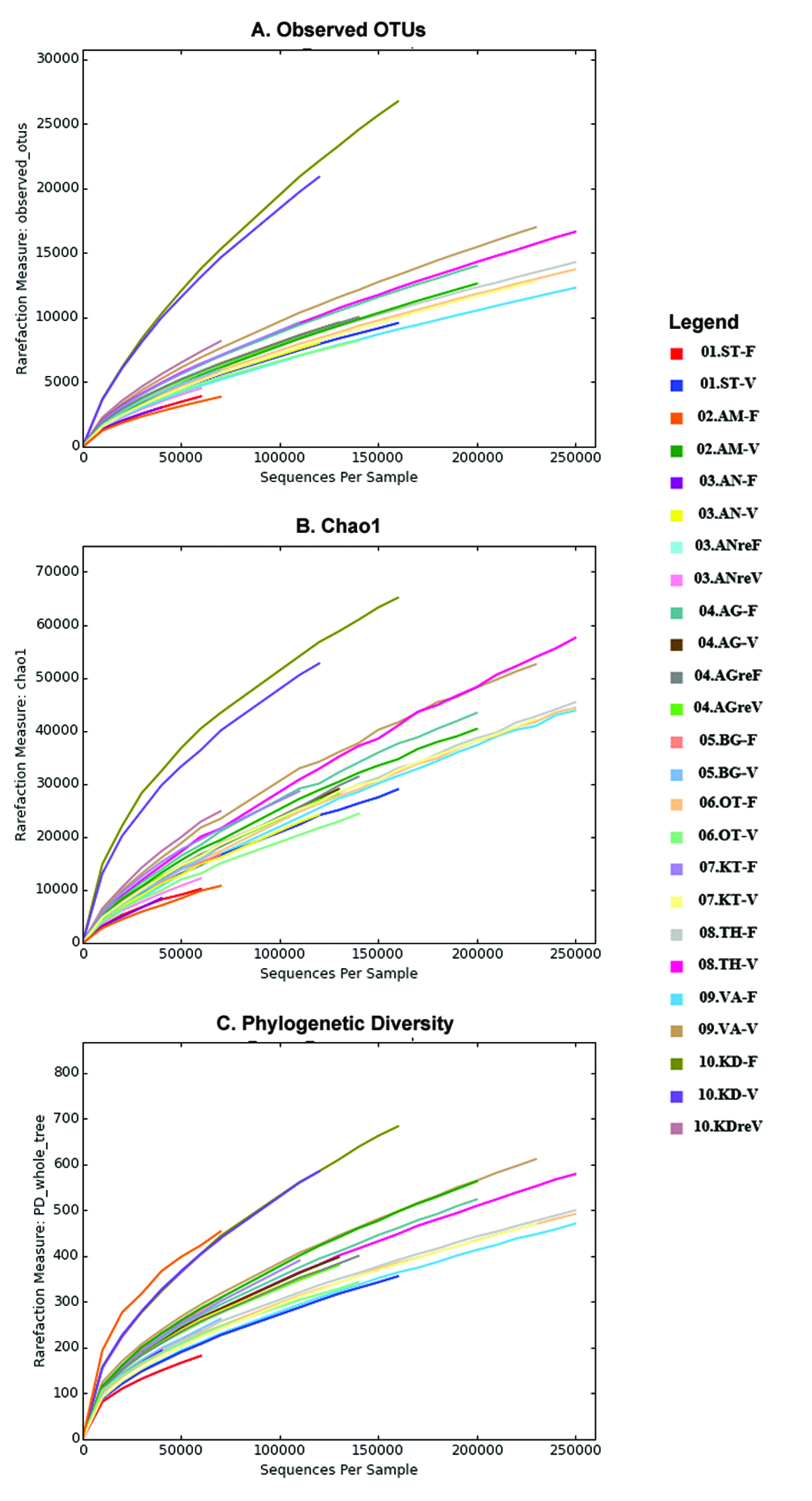

Supplement: Supplementary file 2 [file ECE3-8-6463-s002.tiff]

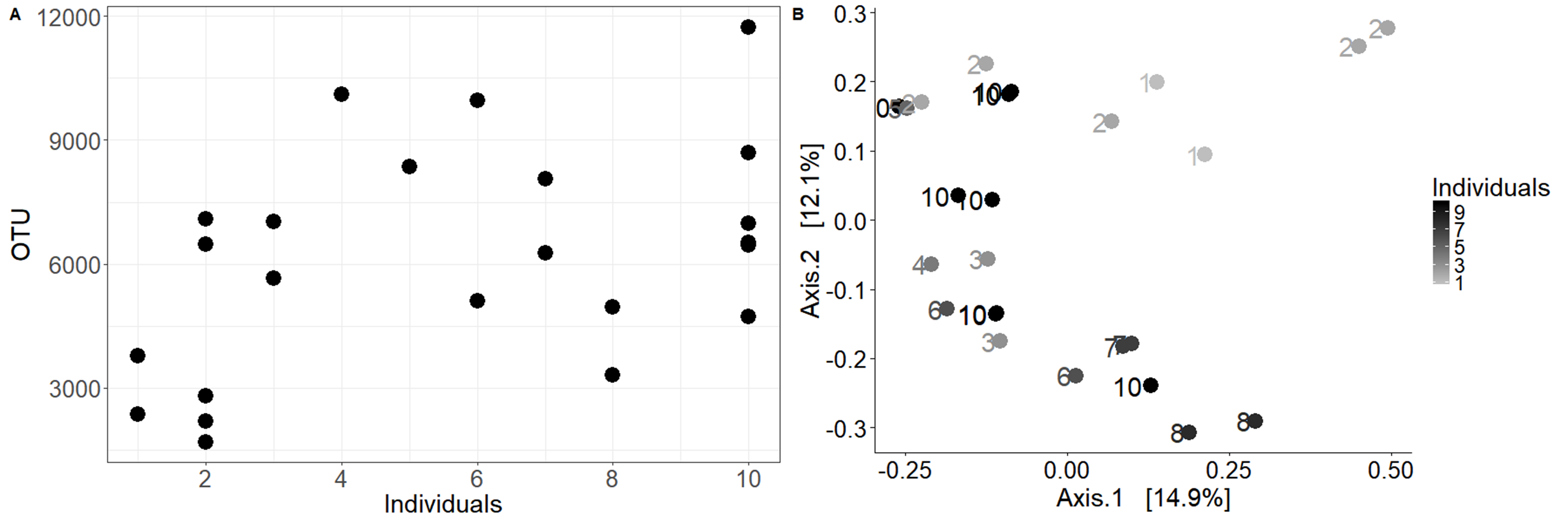

Supplement: Supplementary file 3 [file ECE3-8-6463-s003.tiff]
