# Supplementary material for: Gut microflora may facilitate adaptation to anthropic habitat: A comparative study in Rattus
Source: Ecol Evol. 2018 Jun 14;8(13):6463–72. doi: 10.1002/ece3.4040 (PMC6053588; doi:10.1002/ece3.4040)
Supplement: Supplementary file 4 [file ECE3-8-6463-s004.doc]

# AV_SupMat_Figures: Gut microflora of two *Rattus* species

Figure S 1 Sequencing summary

A) Distribution of reads per OTU, B) Distribution of reads per sample

Skewed distribution of reads per OTU indicates that very few OTUs are over-abundant and most are rare. Even distribution of reads for all samples indicates no sample-bias.

Figure S 2 Rarefaction plots

A) Observed number of OTUs and B) increase with sampling depth indicating that there are probably several spurious OTUs.

Figure S 3 Effect of pooled number of individuals on observed number of OTUs

A) There is no linear relationship between the number of individuals and the log10 of number of OTUs (Spearman’s rank correlation coefficient ρ=0.15, p-value = 0.5). B) Principal coordinates analysis plot (constructed with Bray-Curtis dissimilarity index) indicating that there is no significant effect of the number of individuals on composition of gut microflora.
